# The Genetic Basis of Graves' Disease

**DOI:** 10.2174/138920211798120772

**Published:** 2011-12

**Authors:** Rafał Płoski, Konrad Szymański, Tomasz Bednarczuk

**Affiliations:** 1 Department of Medical Genetics, Centre for Biostructure, Medical University of Warsaw, Poland; 2 Department of Internal Medicine and Endocrinology, Medical University of Warsaw, Warsaw, Poland; 3 Endocrinology Unit, Mossakowski Medical Research Centre Polish Academy of Sciences, Warsaw, Poland

**Keywords:** Graves’ disease, thyroid, autoimmunity, association, HLA, non-HLA genes.

## Abstract

The presented comprehensive review of current knowledge about genetic factors predisposing to Graves’ disease (GD) put emphasis on functional significance of observed associations. In particular, we discuss recent efforts aimed at refining diseases associations found within the HLA complex and implicating HLA class I as well as HLA-*DPB1* loci. We summarize data regarding non-HLA genes such as *PTPN22*, *CTLA4*, *CD40, TSHR* and *TG* which have been extensively studied in respect to their role in GD. We review recent findings implicating variants of *FCRL3* (gene for FC receptor-like-3 protein),* SCGB3A2 *(gene for secretory uteroglobin-related protein 1- UGRP1) as well as other unverified possible candidate genes for GD selected through their documented association with type 1 diabetes mellitus: *Tenr–IL2–IL21*, *CAPSL* (encoding calcyphosine-like protein), *IFIH1*(gene for interferon-induced helicase C domain 1), *AFF3, CD226* and *PTPN2.* We also review reports on association of skewed X chromosome inactivation and fetal microchimerism with GD. Finally we discuss issues of genotype-phenotype correlations in GD.

## EARLY EVIDENCE FOR GENETIC PREDISPOSITION TO GRAVES’ DISEASE – FAMILY STUDIES

Graves’ disease (GD) is an autoimmune disorder in which antibodies activate the thyrotropin receptor (TSHR) causing a hyperfunction of the thyroid gland. This activation stimulates follicular hypertrophy and hyperplasia, leading to thyroid enlargement and increases thyroid hormone production. GD affects approximately 0.5-2% of the Caucasian population and is the cause of the majority of cases of hyperthyroidism. 

The familial occurrence of autoimmunological thyroid disorders (AITD) has been noticed a relatively long time ago when it was reported that a third of siblings of GD patients developed AITD and over half of asymptomatic children had thyroid antibodies in their blood [1]. Similar observations were made when examining patients with Graves’ ophthalmopathy (GO), of which 36% had a first- or second-degree relative affected with GD or other AITDs [2]. 

Perhaps the most convincing evidence for genetic predisposition to the disease is provided by twin studies. While in monogenic diseases there is a full concordance among monozygotic (MZ) twins, in disorders with complex inheritance the concordance is incomplete, but still higher compared to dizygotic (DZ) twins. Such observations were made in several twin studies of GD. In the Dutch twin cohorts the probandwise concordance rates were 0.36 and 0.03 for MZ and DZ pairs, respectively [3]. Model-fitting analysis of these data showed that 79% of the predisposition to the development of GD is attributable to genetic factors whereas individual specific environmental factors not shared by the twins could explain the remaining 21% [3,4]. The results obtained during extensive American twin studies indicated that the estimated pairwise concordance for GD is 0.17 in MZ twins and 0.02 in DZ siblings [5] suggesting a smaller contribution of genes for GD development in American vs. European population.

Family and twin studies clearly demonstrated that GD is not caused by a single gene defect but has a complex pattern of inheritance [6,7]. Today it is clear that genetic predisposition to GD is accounted for by multiple genes with very modest individual effects. The majority of already identified loci confer only low risk for disease (~1.2-1.5) and it is likely that loci with even weaker effects will be discovered in the future. 

## METHODOLOGICAL APPROACHES USED FOR IDENTIFYING GENETIC VARIANTS PREDISPOSING TO GD

The main methodological approaches used for studying genetic predisposition to GD are based on linkage or association analysis.

### Linkage Analysis

Linkage analysis is based on study of family(ies) allowing to evaluate the co-segregation of a genetic variant with disease. If a tested marker is close to a disease-causing variant the frequency of recombination between them may be sufficiently low to cause a preferential inheritance of the marker among affected individuals, even though the marker itself is not involved in disease pathogenesis. The likelihood of linkage between a disease and genetic marker is described by the odds calculated as the ratio of the likelihood of a given distribution of genotypes in a family assuming linkage vs. assuming random segregation. This likelihood ratio is typically presented as a logarithm with the base of 10 and is called LOD (logarithm of odds) score. A significant evidence for linkage occurs when a LOD score is greater than 3.3 and is replicated at least in two separate studies [8]. In a typical approach a set of ~300 markers is sufficient to cover the whole genome so that a study can be performed. Whereas linkage analysis has proved valuable in analysis of Mendelian traits its use for examining complex disorders like GD is limited by the requirement for multiplex families and by its low power to detect weak effects. Another limitation of linkage analysis is low resolution which makes it usually impossible to separate effects of loci within 2-3 Mb. 

### Association Analysis

Association analysis is a more sensitive method to detect weak genetic risk factors than linkage analysis and currently is the predominantly used method. Association analysis is based on comparing the frequency of a variant in patients and in ethnically matched controls. If a statistically significant difference in the frequency of a variant is observed between cases and controls it is concluded that this variant is associated with disease. The strength and direction of an association is expressed as OR (odds ratio) which is a parameter approximately equal to relative risk (RR) if the disease is relatively rare (< 10%) which generally is the case for GD. For biallelic polymorphisms such as single nucleotide polymorphisms (SNPs) it is common to calculate OR so that it reflects the effect of the variant with lower population frequency (MAF or minor frequency allele). Whereas association studies offer better resolution than linkage analysis (~0.5Mb) it still should be emphasized that presence of an association does not imply causality and should usually be interpreted only as pointing to direct involvement of some variant(s) in linkage disequilibrium (LD). Indeed, it is common to observe statistically significant associations of a group of markers whose individual effects cannot be separated due to strong (sometimes absolute) LD [9]. 

Recently, due to advances in high throughput genotyping technologies it has became possible to conduct genome wide association (GWA) studies, i.e. association studies using hundreds of thousands of markers which allow to scan the entire genome and not only candidate loci suggested by prior hypotheses [10]. Implementation of the GWA approach significantly benefitted from information made available through the HapMap project [11]. Within the HapMap 10 million SNP across the entire human genome were typed allowing to dissect patterns of LD and to define haplotype blocks consisting of highly correlated SNPs. Based on this, an informed selection of SNPs to analyze became possible allowing to maximize coverage without unduly increasing costs. 

Despite their unquestionable value GWA studies have limitations such as a potential for false-positive results which necessitates very large sample sizes, genotyping errors or insensitivity to structural variants [12]. Another criticism of current GWA studies stems from the increasingly recognized possibility that in addition to common polymorphisms, which are currently focused on, the disease risk may be significantly influenced by rare (<0.05) or very rare genetic variants (<0.001). For example, recently Surolia *et al.* [13] identified a number of rare/very rare defective variants within sialic acid acetylesterase (SIAE) gene which together were clearly more prevalent among patients with autoimmune disorders than among controls. Although our data do not support the role of *SIAE* in GD [14] this study [13] is a good example of an increasingly popular approach in the field of genetics of complex diseases. 

### Future Approaches – Next Generation Sequencing 

Whereas at present the most interesting novel data on genetics of autoimmune diseases come from carefully designed GWA studies (such a study in GD, not limited to coding variants, is still conspicuously missing!) in the near future this technology may be replaced by even more powerful approaches based on next generation DNA sequencing. Progress in this field makes it possible to sequence genomes or large parts thereof (such as an exome, i.e. all exons from a genome) at unprecedented speed and a wealth of information on rare variants obtained using this technology has already been provided by The 1000 Genomes Project [15]. Whereas at present this effort concentrates on healthy people [15] in the near future similar projects involving patients in addition to controls will most likely be undertaken.

A more detailed discussion of methodological approaches for analysis of genetic predisposition to GD can be found in an excellent recent review [16]. 

## GENES PREDISPOSING TO GD

### HLA Region

A set of genes located on the short arm of chromosome 6 termed the Major Histocompatibility Complex (MHC) has been attracting attention of immunogeneticists ever since it was shown to determine survival of allogeneic grafts. Human MHC is often referred to as the HLA region in parallel with the major loci which are located there and which encode proteins known as Human Leukocyte Antigens. HLA molecules are central for the function of the immune system because they bind fragments of antigens in the form of peptides and present them to T lymphocytes. 

HLA molecules are divided into HLA class I (HLA-A, B, C) and class II (HLA-DR, DQ, DP). HLA class I molecules interact with CD8 lymphocytes which fulfill mainly cytotoxic effector functions. Because all tissues need to be surveyed by CD8 lymphocytes, HLA class I molecules are widely expressed. HLA class II molecules present antigens to CD4 lymphocytes which initiate and regulate specific immune response. Although the final outcome of an encounter with an antigen is determined by many factors, binding of an antigen fragment by HLA class II is necessary for the development of any specific immune response. HLA class II molecules are permanently expressed on the surface of cells involved in antigen presentation (dendritic cells, macrophages, B cells) whereas expression in other cells may be induced by inflammation. Apart from their direct involvement in immunologic reactions HLA molecules are necessary for ontogenesis of the immune system since they participate in the maturation and selection of lymphocytes in the thymus [17].

HLA genes and molecules display a polymorphism which is particularly extensive in regions, known as ‘pockets’, which directly bind peptide residues. HLA polymorphism has profound functional significance because different HLA variants bind a distinct repertoire of peptides. HLA polymorphism results from evolutionary selection driven by advantages associated with a diversification of the pool of variants present in a population. One significant advantage of HLA diversity may be to hinder the spreading of infections.

HLA class I molecules consist of a heavy chain non-covalently bound with beta2 microglobulin (b2m, also called the light chain) which is non polymorphic and is encoded outside the HLA complex. HLA class II molecules are composed of two chains of similar mass (alpha and beta chain) which are encoded by distinct loci. In case of HLA-DQ both chains (encoded by the *DQA1* or *DQB1* loci, respectively) are polymorphic whereas in the case of DR and DP significant polymorphism is limited to the beta chain (encoded by the *DRB1* or *DPB1* loci). Thus, to characterize HLA-DQ, both chains or the appropriate genes need to be analyzed, whereas in the case of DR and DP it is sufficient to analyze the beta chain.

The HLA complex is characterized by strong LD. In Caucasians exceptionally strong LD is found between genes encoding DR and DQ molecules so that presence of a given *DRB1* variant to a large extent determines *DQA1* and *DQB1* alleles and the other way around. Because of this strong LD it is often convenient to refer to HLA haplotypes in order to describe blocks of alleles virtually always found together in a population. It is usually difficult to characterize effects of individual alleles forming a haplotype.

Within the MHC complex the problem of LD is relevant also because in the close proximity of HLA genes there are numerous other loci making MHC the most gene rich region in the whole human genome [18]. Some of these loci encode proteins with accessory functions in antigen presentation such as LMP2/LMP7 and TAP1/TAP2 which function in HLA class I pathway, or DMA and DMB which are similarly involved in the HLA class II pathway [19]. Another group of MHC located loci such as *TNF*, genes encoding complement components or heat shock proteins take part in an immune response without direct interaction with HLA. However, despite clear enrichment for genes involved in immune response, approximately 60% out of 252 expressed MHC loci have other functions [20].

The majority of autoimmune diseases including GD display strong associations with HLA variants [21]. In Caucasian GD patients increased prevalence of the DRB1*03 DQA1*05 DQB1*02 haplotype is usually observed suggesting that some gene(s) encoded there increase the risk of disease development 2-3 times [22]. At present it is not clear which locus is responsible for the observed association. Out of the three most frequently studied genes encoded on this haplotype DQB1*02 can be excluded as it is commonly found on haplotypes encoding DRB1*07 which appear to protect from GD [23-25]. Conversely, the relative importance of DQA1*05 and/or DRB1*03 is under discussion. Although in some studies a stronger association with DQA1*05 was found [26, 27] an argument against a primary role of this allele is the lack of established association between GD and DRB1*11/12 which in Caucasians are also in strong LD with DQA1*05. 

In contrast to the attempts to disentangle LD by statistical methods, an approach similar to the one originally used to develop ‘shared epitope’ hypothesis in rheumatoid arthritis [28] suggested a primary role of the *DRB1* locus in GD [29]. This suggestion was based on direct analysis of DNA sequence of exon 2 of the HLA-*DRB1* gene in 208 GD patients and 149 controls with a focus on amino acid position 74 which was identified as the only polymorphic position among those forming the DR peptide-binding pockets specific for DRB1*03. Whereas variants with arginine at position 74 were expected to be overrepresented among GD patients due to increased prevalence of DRB1*03, the interesting observation was that they were associated with disease also when only DRB1*03 negative subjects were considered (OR=10.5, P= 0.02) [29]. The possibility that DRB1 position 74 is a primary determinant of GD susceptibility was consistent with results of a subsequent study analyzing *DRB1*, *DQB1* and *DQA1* loci in 871 patients and 621 controls, although a conclusive test was not possible because of the small number of subjects in whom Arg-74 was not associated with DRB1*03 [24]. Arg-74 was also found to significantly contribute to the pocket structure conferring risk for HT [30] and was associated with co-occurrence of T1DM with AITD [31]. 

In parallel with susceptibility conferred by Arg-74 it was proposed that Gln-74 protects against GD [29]. This was consistent with the observation that DRB1*07 alleles which always encode Gln-74 are found at decreased frequency among GD *vs.* controls [24,25,29]. Different effects of Gln-74 and Arg-74 on HLA-DR peptide binding were also suggested after structural modeling [31].

A turn in the discussion about the role of HLA in GD was brought about by the suggestion that the disease may be primarily associated with alleles of HLA class I, in particular HLA-C*07 [32]. While studying distribution of alleles of the HLA-*B*,*C*, *DRB1*, *DQA1* and *DQB1* loci among ~500 patients and controls from the UK it was observed that the strongest association was with HLA*C (P=1.20x10^-22^). Whilst the *DRB1* was the next most strongly associated locus, the statistical significance of its effect was distinctly lower (P=6.67x10^-12^) [32]. Analysis of individual HLA-*C* alleles showed that C*07 was the most predisposing allele (OR 1.63) whereas C*03 and C*16 had protective effects. Although the association with HLA-*C* alleles was the strongest other locus/loci had independent effect(s). When genotypes or alleles of other tested loci were added to a model already containing HLA*C variants, an improvement of disease prediction was observed in all analyses with the most pronounced effect for HLA*B. Analyses of individual contribution of HLA*B alleles showed the strongest effect for B*08 and a protective association for B*44 [32]. Both C*07 and B*08 are encoded on the common haplotype associated with GD in Caucasians which also includes DRB1*03, DQA1*05 and DQB1*02. However, the authors concluded that the observed associations to HLA class I alleles could not be attributed to LD within this haplotype [32].

Whereas Simmonds *et al.* clearly demonstrated that in Caucasians in addition to DQA1*05 and/or DRB1*03 there are other HLA linked genes which are associated with GD a limitation of their study was the lack of analysis of HLA-*A* and HLA-*DPB1* loci [32]. Since in Caucasians the search for association between GD and *DPB1* alleles has been relatively limited and inconclusive [33-35], it should be interesting to include this locus in adequately powered future studies. 

The association between GD and HLA loci from the haplotype encoding DRB1*03 DQA1*05 and DQB1*02, although well documented in Caucasians, is conspicuously absent in other ethnic groups. 

The most comprehensive study on HLA associations in GD in an Asian population was recently published by Chen *et al.* [36]. While studying ~500 GD patients and controls selected in a case control design as well as a cohort of families including >400 patients these authors found that HLA-DPB1*05:01 was the major gene predisposing to GD among Han Chinese with an OR=2.3 and a high population-attributable risk of 48%. Other susceptibility variants with independent effects included B*46:01, DRB1* 15:02 and 16:02 whereas DRB1*12:02 and DQB1*03:02 conferred protection [36]. Unfortunately, the authors did not study the *DQA1* locus so that an effect of DQA1*05:01 whose primary role was postulated in Caucasians [22] was not directly evaluated. However, the authors argued that association of DQA1*05:01 with GD in their cohort was not supported by LD patterns observed in Asians [36]. The data from the study of Chen *et al.* did not support the role of Arg-74 as proposed in Caucasians [29].

In addition to the novel observations Chen *et al.* provided an excellent review of previous studies on HLA associations in GD performed among Asian subjects [36]. One interesting conclusion from this review was that the association with DPB1*05:01 had been observed in three out of four former studies which analyzed this locus but had not been appropriately focused on subsequently [36].

Relatively few studies on HLA associations with GD have been performed among Blacks [37-42]. Whereas all these studies were based on limited numbers of patients (n~50) it is noteworthy that only one reported an association with the DRB1*03 variant [38]. Notably, none of the studies analyzed the *DPB1* locus. Since Blacks display the largest HLA diversity, well powered studies of GD in this population may be particularly worth performing in the future.

It is likely that HLA associations with autoimmune diseases including GD are caused by the physiological function of these molecules, i.e. antigen presentation [21]. The frequently postulated hypothesis posits that disease-associated variants but not other variants efficiently bind peptides derived from autoantigen(s) such as thyroglobulin or TSHR and thus have a permissive role in the development of immune response. Another possibility is that disease-associated HLA variants operate at the stage of thymic selection unfavorably influencing positive and/or negative selection of T cell clones with regulatory or effector functions. Additional mechanisms proposed to explain HLA class I association included influence on NK (natural killer) cell repertoire through interactions with killer immunoglobulin-like receptors (KIR) and/or serving directly as a source of autoantigens after misfolding and presentation by HLA class II molecules [32]. It should be emphasized that these mechanisms remain speculative. 

In conclusion, although the HLA region clearly contains genes predisposing to GD, the exact identity of involved variants or even loci remains unknown. There is an agreement that in Caucasians at least some of these genes are located on common DRB1*03 haplotypes whereas in Asians an emerging consensus points to a role of the *DPB1* variants. It should be emphasized that HLA associated risk for GD may well reflect superimposing effects (also protection) of many alleles of different loci including loci encoding other molecules than HLA. Importantly, the variants involved need not be the same in all ethnic groups due to differences in genetic profile of the background population and/or differences in pathogenesis caused for example by different pathogen milieu. 

Clearly high resolution HLA studies across various ethnic groups are needed. Such studies are particularly warranted since the available evidence indicates that similarly as in other autoimmune diseases the HLA linked gene(s) may be the strongest of all genetic factors predisposing to GD. 

### Protein Tyrosine Phosphatase-22 (PTPN22)

Lymphoid tyrosine phosphatase (LYP) encoded by the protein tyrosine phosphatase-22 (*PTPN22*) gene was originally associated with T1DM based on candidate gene approach [43] and subsequently shown to increase the risk for a number of autoimmune diseases including GD [44], rheumatoid arthritis (RA), juvenile idiopathic arthritis (JIA) and autoimmune Addison disease (AAD) [45]. In many autoimmune diseases *PTPN22* represents the second most strongly associated locus after HLA. 

PTPN22 is involved in limiting the adaptive response to antigen by dephosphorylating and inactivating T cell receptor (TCR) associated kinases and their substrates. In lymphocytes, PTPN22 physically associates through a proline-rich motif with the SH3 domain of the Csk kinase, an important suppressor of the Src family kinases that mediate TCR signaling. PTPN22 inhibits activation of T cells in a synergistic manner with Csk [46,47]. 

The best documented association of *PTPN22* variants to autoimmune disorders including GD is rs2476601 (C1858T). The disease-associated C1858T SNP, encoding an Arg to Trp substitution at residue 620 (R620W), is located in the P1 proline-rich motif of PTPN22, which binds with high affinity to the Src homology 3 (SH3) domain of the tyrosine kinase, Csk. The W620 variant disrupts the interaction between PTPN22 and Csk [48] and also increases phosphatase activity, which in turn suppresses TCR signaling more efficiently than the wild-type protein [49,50]. *In vitro* experiments have shown that T-cells expressing the W620 allele may be hyperresponsive, and therefore carriers of this allele may be prone to autoimmunity [43,48]. A role of PTPN22 in T-cell regulation has been confirmed by the results of knocking out the murine homologue of *PTPN22*, which lowered thresholds for T-cell-receptor signaling and inhibited production of IL-2 in these animals [51]. However, in another study, significantly higher numbers of IL-2 producing cells in W620 carriers after autoantigen stimulation have been found, suggesting that the W620 variant is rather a loss-of-function variant [52]. One explanation of these discrepant results is that the decreased levels of IL-2 associated with the W620 variant may be caused, rather than by an increase in activity, by an alteration in the cellular location of PTPN22 which impairs the binding of PTPN22 to the protein tyrosine kinase Csk leading to a phenotypic change [52,53].

Despite these contradictory observations, the net result of the *PTPN22* 620W substitution appears to be a gain of function with respect to reduction of phosphorylation of key signalling molecules and associated downregulation of TCR signaling which leads to inhibition of expansion of T cells, weakening of the positive selection in the thymus, and reduction of antibodies’ production through lowering activity of helper T lymphocytes [51].

Interestingly, there are reports indicating that human PTPN22 also inhibits the activity of B-cell antigen receptor [50,54]. Although it seems counterintuitive that the blunted cell activation should lead to increased risk of autoimmunity, there is evidence for such a mechanism also in other models [55]. While a full explanation of these findings is not yet possible, the dysfunction of regulatory cells and deregulation of lymphocyte maturation in the thymus have been invoked as mechanisms [56]. 

Association between *PTPN22* 620W polymorphism and GD has been demonstrated in numerous studies among Caucasians with strength typically estimated as OR 1.5-1.9 [44,57-59], which makes this variant one of the strongest known genetic factors predisposing to autoimmune diseases. A gene dose-dependent effect of *PTPN22* 'T' allele on the age of onset of Graves' disease has been observed in a Polish population [57] but was not replicated in a cohort from the UK [60].

* PTPN22* has multiple listed copy number variants (CNVs) encompassing the entire gene. When CNVs deposited in a Database of Genome Variants [61] for the *PTPN22* gene were tested, only two controls showed a copy number change, one deletion and one duplication, suggesting that CNVs at this locus do not contribute to the etiology of GD [62]. 

Although most of the studies focused on the C1858T variant, other polymorphisms of the *PTPN22* gene may also be relevant. The variant R263Q has been shown to reduce the risk of systemic lupus erythematosus (SLE) due to reduced phosphatase activity [63]. In a study of patients with GD from the UK population, a haplotype reducing the risk of disease, regardless of the effect of the polymorphism at position 1858, has been identified [64]. The conclusions from this study were generally similar to an earlier one conducted among patients with RA [65]. However, it must be stressed that the protective haplotypes identified in the aforementioned studies were different [64,65]. The role of other variants of *PTPN22* is particularly interesting in populations of Asians and Africans, in which R620W occurs very rarely or not at all [66,67]. The total absence of R620W in patients with AITD was confirmed in the Japanese [68]. However, a subsequent analysis of SNP markers within the region of the *PTPN22* gene revealed the presence of a protective haplotype which is different from those identified previously [69]. 

Overall, available results indicate that apart from the well-known polymorphism C1858T, the *PTPN22* locus contains other functionally important variants, particularly those conferring protection. Currently, the identity of these variants, except for R236Q, remains unknown, and their effect can be estimated only indirectly through analysis of haplotypes which differ in frequency between patients and healthy controls.

### Cluster of Differentiation 40 (CD40)

* CD40* has been associated with GD as a positional candidate on the basis of a genome-wide linkage study in GD which implicated 20q11 chromosomal region, designated GD-2, as harboring a susceptibility locus [70,71]. A potentially functional SNP in the CD40 gene is the C/T polymorphism (rs1883832) located at position -1 relative to translation start and affecting Kozak sequence which plays a major role in the initiation of the translation. The C allele of rs1883832 has been found to confer OR=1.6 for GD among Caucasians whereas the results from *in vitro* transcription/translation system suggested that this allele predisposed to disease by increasing the efficiency of translation of CD40 mRNA [70,71]. 

The association between GD and rs1883832 was not replicated in two large case-control studies in the United Kingdom [72,73]. Despite this controversy [72,73] association between the rs1883832 CC genotype and GD has been confirmed in a meta-analysis including 1961 and 1960 Caucasian patients and controls, respectively, as well as 132 patients and 164 controls from Korea (OR=1.22, 95% CI: 1.08–1.38) [74]. The association between GD and C variant rs1883832 is also supported by studies in the population of Japan although in this population the effect may be limited to patients with late onset of disease [75] and/or to the CC and CT genotypes, suggesting a dominant rather than a recessive model of inheritance [75,76]. 

Even though a CNV in the CD40 gene is listed in the DGV, Huber *et al.* while using two different assays failed to find any deletions or duplications of CD40 locus in either the controls or GD patients [62]. 

CD40 is a surface molecule of the TNFR (tumor necrosis factor receptor) family constitutively expressed on a variety of cells in the immune system, including antigen-presenting cells (APCs) and B cells [77], as well as on other types of cells, for example, thyroid follicular cells [78] and orbital fibroblasts [79]. CD40 plays a fundamental role in B cell activation as its ligation provides the necessary co-stimulatory signal for B cell proliferation, immunoglobulin class switching, antibody secretion, prevention of apoptosis of germinal-center B cells, affinity maturation and generation of long-lived memory cells [80,81]. The physiological ligand for CD40 is the CD154 (CD40L) molecule which is expressed on the surface of activated T helper cells [82]. 

Recently, inhibition of CD40 expression by siRNA was evaluated for potential to prevent disease development in mice immunized with adenovirus expressing human TSHR A subunit. However, despite successful lowering of CD40 expression, no effect on the rate of disease induction was observed [83].

### The Cytotoxic T Lymphocyte-Associated Factor 4 (CTLA4 also known as CD152)

* CTLA4 *consists of four exons encoding different functional domains such as a leader sequence and extracellular, transmembrane as well as cytoplasmic domains. The CTLA-4 protein acts as a potent negative regulator of T-cell response [84,85] and variants of *CTLA4 *have been consistently associated with numerous autoimmune disorders [86].

CTLA-4 acts by delivering an inhibitory signal through its cytoplasmatic domain which can reverse the classic TCR-induced stop signal needed for physical interaction between T cell and APC, thus reducing adhesion periods between these cells which in turn decreases cytokine production and proliferation [87-89]. In addition to this cell intrinsic action another model emphasizing the role of soluble CTLA-4 (sCTLA-4) has been proposed [90]. The mechanism of the cell extrinsic action could involve stimulation of regulatory T cells but the precise mechanism is not clear since elevated levels of sCTLA-4 are found in patients with autoimmunity [91,92]. Recently Omaer *et al.* demonstrated yet another cell extrinsic mode of CTLA-4 action occurring through removal of co-stimulatory ligands (CD86) from APCs *via *trans-endocytosis [93]. Clearly, more work is needed to decipher all physiologically relevant effects CTLA4. 

The most consistent associations with GD within *CTLA4* locus were found with three polymorphisms, the first being the AT-microsatellite polymorphism (ATn) at the 3’untranslated region (3’UTR) of the gene [94,95]. It has been proposed that long AT-repeat allele decreased stability of *CTLA4 *mRNA thus blunting the inhibitory function of the protein and thus reducing control of T-cell proliferation [96]. 

The second polymorphism implicated as causative was rs231775, a SNP (A49G ) in the signal peptide causing a substitution Thr to Ala [95,97-103]. This amino acid change could influence post-translational processing leading to inefficient glycosylation of the autoimmunity predisposing variant [104]. However studies conducted by Hu *et al.* did not support these observations as both the extrinsic and intrinsic actions of recombinant human CTLA-4 expressed in transfected Jurkat T cells were not affected by the signal peptide polymorphism indicating that the association of the rs231775 to GD is secondary to LD with the causative variant [105]. 

Another widely studied genetic *CTLA4 *polymorphism is rs3087243 located downstream from the 3’UTR of the *CTLA4 *and also known as CT60 [101,102,106-108]. Investigation of full-length (flCTLA-4) and sCTLA-4 expression after taking into account the CT60 genotype revealed a lower expression of sCTLA-4 in individuals homozygous for the G allele [109,110]. However, this result was not replicated in a larger Swedish study which also did not find any correlation between concentration of serum sCTLA4 and disease status or CT60 genotype [111]. Recently, a significant association with *CTLA4 *CT60 was found for GD (OR=2.97) in Japanese patients. Stratification analyses suggested a possible synergistic interaction of *CTLA4 *with HLA-A*02 and -DPB1*05:01 in the susceptibility to GD [112]. 

In a comprehensive meta-analysis by Kavvoura *et al.* [113] the A49G and CT60 polymorphisms among Asians and Caucasians have been studied. There were 28 studies that reported data for the A49G polymorphism and 7 for CT60 with a total of 4848 GD cases typed for A49G and 3047 for CT60 as well as 7314 and 3741 healthy controls, respectively. The summary OR for A49G was estimated as 1.49 with respect to G-allele carriers with a significant between-study heterogeneity. Interestingly, the magnitude of the overall OR diminished over time, from 1.64 in 1996 to 1.49 found in meta-analysis by Kavvoura *et al.* in 2007 [113]. The G allele of CT60 polymorphism increased odds of GD 1.45 fold and similarly to A49G, there was a significant heterogeneity among studies [113]. 

The obtained results were similar for Asians and Caucasians but the Asian studies were more divergent [113]. Haplotype analysis indicated that GD association with A49G polymorphism is secondary to LD with CT60 rather then an independent effect. A dose-effect association was observed since carriers of two copies of the risk haplotype had significantly higher odds for disease development when compared to single copy carriers (OR 1.97 vs. 1.29) [113].

Kavvoura *et al.* also analyzed other *CTLA4 *polymorphisms previously described and found a modest association of the G alleles of JO31 and JO30 with GD. No significant associations were obtained for the (AT)n microsatellite, C(-318)T or JO27_1 polymorphism although it should be noted that these polymorphisms were examined in relatively smaller numbers of studies [113]. 

The current state of knowledge does not indicate clearly the causal mechanism behind the association of *CTLA4* with GD. However, *CTLA4* polymorphism is among those most consistently associated with thyroid autoimmune diseases in the majority of populations. 

### The Thyrotropin Receptor (TSHR) 

The thyrotropin receptor (TSHR) is a Gs-protein coupled receptor responding to thyrotropin (thyroid-stimulating hormone, TSH). It is primarily found on the surface of the thyroid epithelial cells [114]. TSH is central to the regulation of the thyroid gland. Since anti-TSHR antibodies in serum are the main serological manifestation of GD, *TSHR* gene was the obvious candidate for genetic studies. *TSHR *is located on 14q31 [115,116] and consists of 13 exons [117]. Initial studies focused on the three common non synonymous SNPs in the *TSHR*: D36H and P52T, both located in the extracellular domain of the receptor, and D727E found in the intracellular part of the protein. Despite the positive results of some studies [118-120], subsequent linkage and case-control studies have largely shown no association of GD with either of these *TSHR* SNPs in Caucasians [121-124]. Nevertheless, genome wide linkage analysis subsequently suggested a GD susceptibility locus in chromosomal region 14q31 [125]. This encouraged extending the search for susceptibility loci to non-coding sequences within the *TSHR* gene. Among the Japanese large scale analyses of SNPs which were prompted by initial associations found by typing of microsatellites [126] showed evidence for three haplotypes within *TSHR *intron 7 that were strongly associated with GD [127]. 

The first evidence for association between *TSHR *and GD in Caucasians has been provided by Dechairo *et al.* [128]. These authors analyzed LD structure across the entire *TSHR *gene, identified LD blocks and analyzed SNPs which captured the majority of intra-block haplotype diversity. One haplotype stretching two LD blocks showed association with GD (OR 1.7), with the most strongly associated SNP (rs2268458) located in intron 1 and conferring OR=1.31 [128]. 

Further refinement of the association between variants in intron 1 of THSR and GD was provided by studies in a UK population by Brand *et al.* who investigated a combined panel of 98 SNPs, including rs2268458, across an extended 800 kb region of the *TSHR *[129]. Among 28 SNPs associated with the disease the strongest signal was at rs179247 (OR=1.53) and rs12101255 (OR=1.55) [129]. The multiple logistic regression suggested these two SNPs in strong LD explained the association signal in the region [129]. Functional analyses suggested that rs179247 and rs12101255 could be associated with reduced expression of full length *TSHR* mRNA relative to two truncated splice variants which in turn could lead to increase in shedding of a part of the *TSHR* receptor called the A-subunit [129]. Since the TSHR A-subunit may be a particularly important target for autoantibodies against TSHR [129,130] its excessive production could well have relevance for GD pathogenesis. 

The results obtained in the UK population were recently replicated in a Polish and extended British cohorts [131]. It was found that rs179247 and rs12101255 were in strong but incomplete LD (D'=0.98 and r^2^=0.50). Logistic regression indicated that association at rs179247 may be secondary to rs12101255 or that rs12101255 is in stronger LD with the etiological variants within the region [131]. Genotype-phenotype correlations provided no clear evidence of association between rs12101255 and any specific clinical characteristics of GD [131].

Although the most strongly associated subregion of *TSHR *gene is different in Caucasians (intron 1) [128,129,131] and Asians (intron 7) [127] there may be some sharing of genetic susceptibility factors between these populations. A marginal (P=0.026) association to an intron 1 SNP (rs2268474) was observed by Hiratani *et al.* in a Japanese cohort [127] whereas another SNP in intron 1 – rs2239610 – was associated with GD in a study of an ethnically mixed Asian population from Singapore [132]. Notably, rs2239610 is a perfect proxy of rs2268458 which was associated with GD in Caucasians [128,129]. On the other hand rs3783941, a nonsynonymous SNP in the distal part of the gene (exon 9) has been associated with GD in a large GWAS study of nonsynonymous variants, albeit only after the control group was expanded to include a total of 4500 subjects [133]. As this association was not replicated, it could be a false positive, although there remains a possibility that the lack of replication was due to insufficient power [129].

To summarise, whereas it is safe to conclude that the *TSHR *gene polymorphism is associated with GD, further fine mapping, perhaps using next-generation sequencing, as well as functional studies will be required to determine the exact location of the etiological DNA variants and to determine to what extent *TSHR* linked susceptibility factors for GD differ between Caucasian and Oriental populations.

### Thyroglobulin (Tg)

Thyroglobulin (Tg) represents one of the major autoantigens for autoimmune thyroid diseases (AITD), including both GD and Hashimoto’s thyroiditis (HT). However, in GD the main autoantigen is TSHR and antibodies against TSHR (TRAb) are found almost exclusively in patients with Graves’ hyperthyroidism. In contrast to TRAb, autoantibodies against Tg (TgAb) are not disease specific and they are found in 80-90% of HT patients, in 50-70% of GD patients (usually in lower concentration) and in other forms AITD. The induction of experimental autoimmune thyroiditis in mice using Tg as an antigen provides further evidence of their major role in the pathogenesis of HT (and not GD) in humans. 

The TG gene has been mapped to chromosome 8q24, which has been linked to HT, AITD (GD and HT) or thyroid antibody production in independent studies [125,134,135]. Detailed sequence analysis of the entire TG gene revealed several intronic and exonic SNPs. One SNP cluster in strong LD (in exons 10–12) and a SNP in exon 33 have been analyzed in further association studies in different populations. There is some evidence that TG polymorphisms are associated with general susceptibility to AITD and separately to HT [136-141]. In several studies, association of TG polymorphisms with GD as well as with the relapse of Graves' hyperthyroidism after antithyroid treatment was found [142-146]. Unfortunately, these results have to be interpreted with caution because of rather small sizes of studied groups. Moreover, the association of TG SNPs with AITD, GD or HT could not be uniformly replicated [147]. Possibly the observed inconsistencies reflect differences in the autoimmune response to Tg in various forms of AITD. Therefore, genetic studies so far support the notion that TG may be a susceptibility gene for AITD in general, and not a specific marker for GD or HT. 

There are several possible mechanisms by which TG SNP’s could influence susceptibility to AITD. Tg is a large glycoprotein dimer, each subunit being ~330 kD. The extent of iodination, changes within the amino acid sequence and posttranslational modifications of Tg may lead to changes in antigenicity and binding to HLA. Indeed, experiments in human class II MHC-transgenic (‘humanized’) mice lacking their own class II MHC genes suggest an important role of human DR3 and DQ8 alleles in initiating Tg-induced experimental thyroiditis [148-150]. Especially exon 33 SNP causing a change from a hydrophobic amino acid (Trp) to a positively charged hydrophilic amino acid (Arg) would be expected to change the structure of Tg. This is in agreement with evidence of the interaction between exon 33 TG variants and HLA-DRB1*03 (Arg74) which increases OR for GD up to 16.1 [137,151]. These results imply that a gene-gene interaction between an immunoregulatory gene (HLA-DR) and a thyroid-specific autoantigen gene (TG) may play a role in susceptibility to AITD. 

Most recently, a newly identified TG promoter SNP (-1623A/G) was associated with AITD [152]. *In vitro* studies suggest that the disease-associated G allele confers increased promoter activity through the binding of the interferon regulatory factor-1 (IRF-1), a major interferon-induced transcription factor. These data fit with observations that interferon-α treatment of chronic hepatitis C infection may be a trigger of AITD and provide a new mechanism of gene-environment interaction in inducing thyroid autoimmunity [152]. Finally, an interesting hypothesis which has not been studied posits that differences in transcription of TG in the thymus could influence the negative selection of autoreactive T-lymphocytes. 

### FCRL3 (FC Receptor-Like-3)

* FCRL3* is a receptor of unknown function with structural homology to classical receptors for immunoglobulin constant chains (Fc receptors). The allele C of rs7528684 located at position –169 in promoter of *FCRL3* (FC receptor-like-3) gene was convincingly associated with GD (OR=2.15, P = 0.00000085) in a Japanese population [153]. *FCRL3* is expressed in lymphoid organs, particular strongly on the surface of the B-cells [153]. Presence of *FCRL3* was also demonstrated on the surface of a subset of T_reg_ cells characterized by lower relative response to antigenic stimulation and reduced suppressor activity [154]. In the original report, rs7528684 was suggested to have functional significance as the disease-associated C allele increased affinity for NFKB transcription factor and showed enhanced transcription rate in luciferase assay [153]. 

The association between GD and *FCRL3* was replicated in two studies in a UK population. Simmonds *et al.* studied 1056 GD patients and 864 controls and reported a difference in allele distribution with statistical significance of P=0.024 [155] whereas The Wellcome Trust Case Control Consortium in an analysis including 2500 GD cases and 2500 controls found an association at rs3761959 (a perfect proxy of rs7528684 according to HapMap) which was significant at P=0.0094 [133]. Notably, in this study, a stronger association (P=1.6x10^-5^) was found with another SNP (rs11264798, located in the intron 8 of *FCRL3*) [133]. Yet another study in a UK population based on 625 cases and 490 controls failed to observe an association at rs7528684 providing instead some evidence for association at rs2282284 in exon 14 (P=0.02 before correction for number of comparisons) [156]. 

In contrast, negative conclusions regarding association of rs7528684 as well as three other SNPs in *FCRL3* with GD were reached by Gu *et al.* who studied 436 cases and 316 controls from a Chinese population. In the case of rs7528684 the conclusion was particularly strong since an opposite effect (protection) was reported. Unfortunately, interpretation of these data is complicated since for rs7528684 the authors present distribution of A/G rather than C/T alleles presumably reflecting a nomenclature based on the other DNA strand than in the original study [153]. However, assuming that A corresponds to T and G to C the data presented by Gu *et al.* are at odds with their conclusion being instead consistent with increased prevalence of CC and CT genotypes among cases vs. controls [145].

Recently, the association to the *FCRL3* region was fine mapped in 2504 patients and 2688 controls from the UK [157]. Although the study confirmed association of rs3761959 (tagging rs7528684) with GD, this association turned out to be secondary to the effect of two other SNPs within *FCRL3*: the previously implicated rs11264798 [133] and rs10489678 [157]. Further analysis suggested that rs11264798 may not be the etiological variant, as the effect of risk G allele of this SNP was haplotype dependent being apparent only when present together with the rs10489678 C allele. 

Overall the available data suggest that genetic polymorphism(s) modifying susceptibility for GD do exist in the *FCRL3* region but the primarily associated variant(s) remain(s) to be found [157].

### Secretoglobin 3A2 (SCGB3A2) Gene Encoding Secretory Uteroglobin-Related Protein 1 (UGRP1)

Variants in the promoter of the *SCGB3A2* gene encoding secretory uteroglobin-related protein 1 (UGRP1) have been associated with GD in an extensive study of a total of ~2500 patients and controls from the Chinese population by Song *et al.* [158] who aimed to explain signals in the chromosomal region 5q12-q33 obtained in previous studies using linkage analysis [134,159]. The strongest association reported by Song *et al.* was to rs1368408 (−112G/A, OR=1.28, P=1.43x10^-6^) and SNP75 (-623~-622 AG/-T, OR=1.32, P=7.62x10^-5^) [158]. Association between GD and the A allele of rs1368408 (OR=1.18, P=0.007) was independently confirmed in a similarly sized UK cohort (SNP75 was found to have low polymorphism and was not analyzed) [160]. Recently, further evidence for association between rs1368408 and GD was provided by a study in a Russian cohort (~1500 cases and controls, OR=1.33, P=2.9×10^-5^) [161]. It should be noted that a relatively early study in a Chinese population did not observe the effect of rs1368408 although this might have been caused by low power due to limited numbers of subjects (~200 cases and controls) [162].

The −112G/A variant of *SCGB3A2* (rs1368408) was originally discovered by Nimi *et al.* who also showed that it was functional with the A allele conferring a 24% reduction of promoter activity [163]. This was confirmed and extended by Song *et al.* who showed that lower expression from haplotypes encoding rs1368408 A was associated with lower concentration of *SCGB3A2* mRNA also in thyroid tissue [158]. Further support for functional significance of the rs1368408 A variant comes from its association with reduced concentration of UGRP1 in serum of healthy subjects as well as of patients with GD or asthma [161,164]. The functional effect of the A allele of rs1368408 may occur through disruption of the binding site for CCAAT/enhancer-binding protein α (C/EBPα) which positively regulates transcription of *SCGB3A2* [158,165].

UGRP1 is a ligand for macrophage scavenger receptor with collagenous structure (MARCO) [166] which has an important function in the innate immune system of the lung where it binds inhaled particles including microbial pathogens and facilitates their clearance by the macrophage system [167-169]. Accordingly, UGRP1 is predominantly expressed in the lung although a low level expression was also found in human thyroid and kidney [158,163]. 

At present it is not clear how variants in *SCGB3A2* predispose to GD. Whereas expression of this gene in the thyroid does not preclude a local effect the well documented function of MARCO (and presumably UGRP1) in lung physiology suggest that the association may be caused by systemic effects originating from the respiratory system. Interestingly, rs1368408 A has been associated with asthma [163]. Although this was not confirmed, an intriguing link between this variant and total IgE, although limited to healthy controls, was recently suggested [161].

Finally it should be noted that other functionally attractive candidates for association with GD such as cytokine genes: *IL3*, *IL4*, *IL5*, *IL9*, *IL13* and the *ADRB2* gene encoding beta-2-Adrenergic receptor are located on chromosome 5q, close to the *SCGB3A2* locus. Notably, out of these, variants in *IL3* [170,171], *IL5* [171] and *ADRB2* [172,173] were all associated with GD. 

### Other Candidate GD Susceptibility Genes 

Because GD is known to share genetic susceptibility with T1DM, a set of SNPs associated with T1DM has been tested in GD (2200 patients, 3600 controls) [174]. Out of 14 SNPs analyzed, seven showed association, although evidence was modest (0.0018<P<0.026) [174]. 

The strongest of associations found was to rs17388568 although the direction of effect (protection) was opposite to that found in T1DM [174]. The rs17388568 is located in chromosome region 4q27 (*Tenr–IL2–IL21*) containing, among others, the locus for IL2. Given the central role of IL2 in immune response it is perhaps not surprising that this region was also associated with other autoimmune diseases such as celiac disease [175], ulcerative colitis [176], RA [177], JIA [178], psoriasis and psoriatic arthritis [179] and others [180] although in the majority of studies another SNP (rs6822844), only weakly linked with rs17388568, was implicated with the minor allele generally conferring protection [181]. It is indeed possible that the 4q27 contains more than one susceptibility locus since rs6822844 but not rs17388568 is associated with RA [181]. In the context of these data, a comprehensive analysis of 4q27 SNPs in GD seems warranted.

The second strongest association reported by Todd *et al.* was with rs1445898 encoding a non-synonymous aa change (Gln75Arg) in calcyphosine-like protein (CAPSL) which possibly has a role related to calcium binding [174]. This finding was not secondary to the effect of an established risk marker of multiple sclerosis (MS) located in the same region (rs6897932 in IL7R) [182-184] as this SNP was not associated with GD [174]. 

The association between GD and rs1990760 [A946T in *IFIH1* -interferon-induced helicase C domain 1 also known as melanoma differentiation-associated 5 (MDA-5) or Helicard] on chromosome 2q24.3 observed by Todd *et al.* [174] was actually reported earlier in another British cohort including 602 patients and 446 controls [185]. A subsequent study in a German population (258 patients, 227 controls) did not report statistically significant association between rs1990760 and GD although the expected trend was noted [186]. No association between rs1990760 and GD was found among the Chinese (261 patients, 206 controls) [187] or the Japanese (290 patients and 229 controls) [188]. 

The association between autoimmunity and rs1990760 was first found in a GWA of nonsynonymous SNPs in T1DM with the minor allele (threonine) conferring protection [189]. The association with T1D was confirmed by a recent meta-analysis (allelic OR= 1.176) although with evidence for some (P=0.023) heterogeneity among studies [190]. rs1990760 and/or other SNPs in the region of *IFIH1* were also associated with MS [191,192], RA [193] (although there is controversy here) [194], psoriasis [195] and, tentatively, with SLE [196].

Whereas statistical analyses did not prove that *IFIH1* was the primary associated locus, support for this hypothesis came from massive resequencing of *IFIH1* in T1DM patients and controls which revealed four rare variants (rs35667974/Ile923Val, rs35337543/IVS8+1, rs35744605/Glu627X, rs35732034/IVS14+1) associated with the disease, independently both from each other and from rs1990760 [197]. 

IFIH1 is one of a family of intracellular proteins involved in innate immunity through recognition of viral RNA [198]. Interestingly, available data indicate that a normal (i.e. encoded by the more prevalent allele/haplotype) activity of IFIH1 increases the disease risk whereas rare variants confer protection. Whereas the rare variants identified by Nejentsev *et al.* were associated with a decreased/absent protein activity, the functional consequences of variation are less clear for rs1990760 since the Thr946Ala change did not influence double stranded RNA binding or *IFN* gene activation [197,199]. It has been shown that Ala946 is associated with reduced *IFIH1* transcription [200,201] but there are also reports conflicting with this conclusion [194,202].

Association to rs9653442 on 2q11 [174] may indicate the role of locus *AFF3* which encodes nuclear protein LAF-4 with homology to a protein involved in leukemia development and a possible function in lymphoid ontogenesis [203]. Through a perfect proxy SNP (rs1160542) rs9653442 has also been associated with RA and JIA [178,204]. 

Another novel association in GD suggested by Todd *et al.* was to rs763361 - a nonsynonymous SNP (Thr307Ser) in the intracellular tail of the CD226 molecule (DNAX-accessory molecule-1 (DNAM-1)) which is a surface receptor with a co-stimulatory function [174]. CD226 is expressed in cells of haematopoetic lineage such as CD4+ and CD8+ T lymphocytes, platelets, as well as some B cells, and natural killer (NK) T lymphocytes [205-207]. Apart from the link with T1D and the tentative association with GD [174] rs763361 has been associated with MS [208], RA [208], SLE [209], Wegener's granulomatosis [210] and systemic sclerosis [211].

The 307Ser variant alters splicing of the CD226 transcript and this has been suggested to explain the association [174]. Another not exclusive possibility is that the amino acid change increases receptor signaling by strengthening the phosphorylation of neighboring sites [208,209]. Conversely, recent *in vitro* analysis indicated a direct functional effect of another SNP closely linked with rs763361 (rs727088), with the disease associated variant causing a decreased expression [208,209]. Consistent with this was the *in vivo* observation that disease-associated rs763361-rs727088 haplotype correlated with lower surface expression of CD226 in T cells and NKT (Natural Killer T) cells [209]. Since no such genotype-dependent differences in expression were observed in NK or B cells it was suggested that CD226 function in T cells or NKT cells was important in disease pathogenesis [209].

Independent associations with GD were also found with two SNPs (rs1893217 and rs478582) in the region of *PTPN2* [174]. *PTPN2* was initially associated with T1DM, Crohn’s disease and RA in a study by Wellcome Trust Case Control Consortium (WTCC) including ~3000 shared controls and sets of ~2000 patients with common diseases including the abovementioned autoimmune disease [212]. These results were later extended to ulcerative colitis [213], JIA [214] and possibly celiac disease [215]. 

Whereas the originally reported association between *PTPN2* and autoimmunity was to rs2542151, the SNPs which were tested and found associated with GD (i.e. rs1893217 and rs478582) were those which showed stronger association to T1DM than rs2542151 [174]. Subsequently it was suggested that there was even stronger association with T1DM at rs45450798 so that this marker should replace rs1893217 [215] but so far there have been no data on association with GD of rs1893217 or SNPs in the *PTPN2* region other than rs1893217 or rs478582. 

* PTPN2* encodes a classical, non-receptor protein tyrosine phosphatase, related to but distinct from PTPN22 which is a very well established risk factor for autoimmunity (see above). *PTPN2* is highly expressed in hematopoietic cells and the phenotype of the knockout mouse model indicates its important role in immune system development, function and predisposition to autoimmunity [216]. 

Apart from the loci associated with T1DM [174] and discussed above, a number of other genes such as *VDR* (vitamin D receptor) [217,218], *FOXP3* (a gene whose expression determines T_reg _commitment), *DIO2* (type II iodothyronine deiodinase), [119], *IL23R *(IL23 receptor) [219] have been associated with GD, based on candidate gene approach (reviewed in [16]). 

In addition to the loci discussed above, a recent study from our group suggested that the A allele of rs4986938 within *ESR2* (estrogen receptor beta) locus was associated with GD, in particular among DRB1*03-negative subjects [220]. Subsequently, rs4986938 was associated with SLE [221] and differences of *ESR2* mRNA concentration suggesting that increased expression of this gene may predispose to autoimmunity [222]. 

We also reported an association between *NFKB1*-94del ATTG (a promoter variant of a gene encoding nuclear factor-κB) and GD which was found in two independent Polish cohorts [P=0.00005; OR=1.37 (1.18-1.60)] [223]. Although the association with susceptibility to GD could not be replicated in a Japanese cohort, subgroup analysis in Japanese GD patients revealed a correlation between the *NFKB1* genotype and the development of GO as well as the age of disease onset [223]. *NFKB1*-94del ATTG has been associated with a decreased transcription [224] and its prevalence was assessed in cohorts with various autoimmune diseases although with variable results [225]. These observations, together with the broad role of nuclear factor-κB in immune function [226], make *NFKB1*-94del ATTG an interesting candidate for further studies in thyroid autoimmunity. 

### Skewed X Chromosome Inactivation (XCI)

In female cells, one of the two X chromosomes is inactivated in early embryonic life and this epigenetic system assures that men and women have equal expression of the genes from the X chromosome, despite the difference in X chromosome copy number. The X chromosome inactivation (XCI) has been regarded a random process which on average leads to inactivation of the maternal and paternal X chromosomes in 50% of cells, respectively. However, in accordance with normal distribution, this random process sometimes generates an unbalanced inactivation pattern with one copy of the X chromosome preferentially expressed. 

Skewed XCI could also result from a bias in the initial choice of the X chromosome which is inactivated due to germline XIST (X-inactive-specific transcript) mutations [84] or deleterious X-linked mutations, X chromosome rearrangements, ageing, twinning, or monoclonal expansion of cells [227]. Importantly, the degree of XCI skewing may also be determined by polymorphic variants on the X chromosome, in particular those located close to the X inactivation centre or chromosomal bands Xq25-q26 [228-231]. Significant skewing of XCI has been arbitrarily defined as a situation in which 80% or more of the cells inactivate the same X chromosome [232].

The original study linking XCI with GD was based on 32 pairs of female twins with AITD (including 19 with GD) and 96 healthy female twin individuals [233]. The study demonstrated a higher prevalence of skewed XCI in blood cells of females with AITD compared with controls. Moreover, skewed XCI was more frequent in female twins with AITD than in their healthy co twins [233].

These results were confirmed by three studies in other populations [234-236]. Ozcelik *et al.* demonstrated skewed XCI in peripheral blood mononuclear cells in a significant proportion (34%) of female subjects with AITDs and 8% of control females [234]. The effect was even more pronounced when extreme patterns of XCI skewing (90:10 or more) were considered – in such an analysis it was found that 20% of AITDs patients showed skewing, compared with only a few percent of control subjects. Skewed XCI (73:27) was also demonstrated in thyroid specimens [234]. Yin *et al.* in a study based on 87 patients with GD, 47 patients with HT and 69 controls confirmed that XCI skewing was associated with both GD and HT (OR = 4.0) [235]. Similar conclusions were reached by Chabchoub *et al.* based on a study of 58 GD patients, 87 HT patients and 257 controls [236]. 

The explanation of the mechanism by which skewed XCI can lead to autoimmunity has been based on the loss of mosaicism hypothesis which posits that lower expression of self-antigens from one X chromosome may interfere with induction of tolerance in the thymus [237-239]. The inefficient negative selection in the thymus could result from the fact that the cells mediating it (i.e. the thymic epithelial and dendritic cells) are derived from a few progenitors (~1 and ~10, respectively) which makes expression of antigens exclusively from one X chromosome possible, especially in the presence of skewed XCI [238]. Owing to an overlap between HLA class I and II antigen presentation pathways these untolerized T cells could in turn provide help to a population of B cells expressing these antigens, thus causing their polyclonal activation. Whereas originally proposed to explain pathogenesis of SLE [238] this mechanism could conceivably decrease the threshold also for other forms of autoimmunity, including GD. 

Another mechanism of autoimmunity development could be related to differences in the degree of XCI skewage found among different tissues [232]. In particular, it could happen that antigens with low expression in the thymus are relatively highly expressed in peripheral tissues (such as thyroid) and induce autoimmunity [237-239]. 

Skewed XCI may offer an interesting explanation for a marked female preponderance of AITD [233]. However, this explanation is difficult to reconcile with the lack of skewed XCI observed in other autoimmune disorders with female preponderance such as MS, SLE or primary biliary cirrhosis [240-242] as well as data from animal experiments showing that female predisposition to autoimmunity is not influenced by homozygosity for X chromosome which should eliminate defective tolerance induction due to skewed XCI [236,243]. 

Although the association between skewed XCI and AITD has been found repeatedly [233-236] its mechanism should be regarded as speculative. Recently the same group who described the original association between AITD and skewed XCI provided preliminary evidence of its noncausality [244]. The argument was based on lack of correlation of within-pair differences in values of XCI with differences in concentration of autoantibodies against thyroid peroxidase observed in MZ but not DZ twin pairs [244]. Whereas the study had limited power mainly because of high within-pair similarities in the concentration of autoantibodies among MZ, its results were interpreted to suggest that skewed XCI and AITD both share genetic determinants but are not causally related [244]. 

Since the degree of XCI skewing may be linked with markers on the X chromosome [231] such an interpretation would suggest that there are as yet unidentified AITD susceptibility loci located on the X chromosome. The relevance of the X chromosome for AITD susceptibility is supported by findings of increased disease risk in Turner’s syndrome, in particular among females with isochromosome Xq, i.e. an X chromosome with deletion of the short arm and duplication of the long arm [245] as well as by observations of increased prevalence of X chromosome monosomy in T and B lymphocytes of females with AITD compared with healthy women [246]. The existence of GD susceptibility loci on the X chromosome was suggested by early studies based on linkage analysis, but without agreement regarding the exact location [247,248]. It should be interesting to look for X linked loci when a GWA study is eventually completed in GD. 

### Fetal Microchimerism (fMC)

Fetal microchimerism refers to presence within the maternal organism of a small population of cells originating from the fetus. fMC arises during pregnancy and may persist for years or decades [249]. A convenient method to study fMC consists of looking among females for male cells or their DNA [249]. 

Since the presence of foreign cells can potentially cause immune deregulation it has been hypothesized that fMC may be a risk factor for AITD and also explain its female predominance as well as frequently observed association between disease onset/aggravation and the postpartum period [250,251]. A first link between fMC and thyroid pathology was provided by demonstration of male cells in thyroidectomy specimens from female patients but not in control autopsy samples [252]. Interestingly, whereas fMC accompanied various thyroid pathologies it was most prevalent in women with HT (5/6 or 83%) [252]. Association between HT and fMC was also found by Klintschar *et al.* who detected fMC in 8/17 patients vs. 1/25 controls [253]. Subsequently, the same group extended these findings using a different methodology [254]. Studying fresh-frozen specimens Ando *et al.* found high prevalence (6/7) of fMC in patients with GD *vs.* controls with thyroid adenoma (1/4) [255]. Somewhat lower prevalence of fMC (4/26 *vs.* 0/6, respectively) was obtained in the same study from paraffin-embedded material which might have suffered from degradation [255]. The association between HT/GD and fMC was also confirmed in a relatively large study of 25 women with HT, 15 with GD and 9 with thyroid adenomas in which prevalence of fMC was 60%, 40% and 22%, respectively for HT, GD and controls [256]. 

Whereas association between fMC and AITD is well documented, it is not clear whether fMC causes the thyroid disease or *vice versa.* Some indirect support for a causative role of microchimerism in AITD has been provided by an observation that twins from opposite sex pairs have a higher frequency of thyroid autoantibodies then monozygotic twins [257]. However, in this setting, the relevant microchimersim should result from *in utero* cell trafficking between twins rather than from migration of fetal cells into the mother organism and thus could be qualitatively different [257]. 

In a murine experimental autoimmune thyroiditis model it was shown that inflamed (but not healthy) thyroid accumulated fetal cells, including T cells and dendritic cells [258]. Although these cells could theoretically contribute to postpartum exacerbation of thyroid inflammation [251,258,259] these data are compatible with high prevalence of fMC in human AITD being a consequence rather than the cause of disease. Indeed, the hypothesis that presence of fetal cells was associated with the maternal response to injury (inflammatory or other) as opposed to causing disease was proposed relatively early [260] and is consistent with observations in animal models [261-263] and humans [264]. 

Furthermore, if fMC were a cause of AITD, an epidemiological association between thyroid autoimmunity and history of pregnancy/parity would be expected. Whereas such an association was indeed found in one cohort [265] it was not observed in four other studies [266-269]. 

Thus, despite association with AITD, fMC is most likely not a risk factor for disease development. Whereas the role of fMC in postpartum disease aggravation cannot be excluded, available data suggest that this and other sex related features of GD such as female preponderance will perhaps be more readily explained by hormonal differences between females and males. Interestingly, in pathogenesis of AITD a role for hyperestrogenic state due to a longer reproductive span has been proposed [266]. The role of hormonal factors in thyroid autoimmunity is also consistent with the association discussed above between GD and a *ESR2 *variant [220]. 

A broader discussion of the role of fMC in autoimmunity and other diseases such as cancer can be found in a recent review [270].

## GENOTYPE-PHENOTYPE CORRELATIONS IN GD

GD is typically characterized by hyperthyroidism, diffuse goiter and the presence of stimulating TSH receptor autoantobodies (TRAb). The severity of thyrotoxicosis in GD is variable and the response to anti-thyroid drugs is difficult to predict. Graves’ ophthalmopathy (GO) is the most common extrathyroidal manifestation of GD. GO significantly impairs the quality of life of affected patients and the most severe cases can be threaten sight. Although the genetic predisposition to GD is well established, the significance of genotype-phenotype correlations remains controversial. Moreover, the lack of complete phenotypic concordance in MZ pairs strongly indicates that environmental and/endogenous factors are of importance [271]. The major environmental factor influencing the clinical course of GD is cigarette smoking.

Several genes have been shown to confer susceptibility to GO (Table **1**). Unfortunately, the results of these studies have to be judged very carefully [272]. It is now apparent that “reliable” association studies should consist of large datasets, small P values, functional polymorphisms and an independent replication. The vast majority of studies analyzing genetic susceptibility to GO are small and underpowered, resulting in a high risk of both false positive and false negative results. Replication studies are lacking or were unable to confirm the initial association between immunoregulatory genes polymorphisms and GO. Finally, the studied groups were usually poorly characterized and the definition of “clinically evident ophthalmopathy” varied between studies. 

Similarly, some studies suggested an association between candidate genes polymorphisms and the course of hyperthyroidism (Table **2**): the age of onset of GD, the relapse rates after anti-thyroid drug treatment and/or the level of anti-thyroid antibodies, with the same limitations as above. Most studies suggested an association between *CTLA4* SNP (A49G) and severity of Graves’ hyperthyroidism. However, these findings could not be uniformly confirmed [273-275]. 

At present, while some genetic differences between subgroups of GD patients have been identified, none of the polymorphisms justifies genetic testing to guide therapy or preventive strategies. Genotype-phenotype correlations in GD remain to be elucidated in the future, through well designed and appropriately powered studies. 

## NOTE ADDED IN A PROOF

While this article was in press a genome-wide association study of GD in a Chinese population was published by Chu *et al.* (Nature Genetics 2011; 43: 897). The study confirmed four previously reported loci (HLA, TSHR, CTLA4 and FCRL3) and identified two new susceptibility markers (rs9355610 and rs6832151). 

## Figures and Tables

**Table 1.** Selected Genetic Markers Associated with Susceptibility to Graves’ Ophthalmopathy

Locus	Polymorphism (allele)	Population [Ref.]	Studied groups	P-value, odds ratio
**Immune- modifying genes: cytokines**				
IFN-γ	intron 1 (CA) repeat	Canada and German [276]	GO (n=53), GH (n=149)	P<0.00001, OR=4.8 For allele IFN-Y*3
TNF	C-863A	Japan [277]	GO (n=62), GH (n=111)	P =0.003, OR=2.7
TNF	T-1031C	Japan [277]	GO (n=62), GH (n=111)	P=0.0003, OR=3.3,
TNF	G-238A	Poland [278]	GO (n=106), GH (n=122)	P=0.008, OR=0.1
IL-23R	rs2201841	USA [219]	GO (n=103), GH (n=111)	P=0.008, OR=1.8
IL-1A	C-889T	Iran [279]	GO (n=50), GH (n=57)	P=0.006, OR=2.16
IL-5	rs2069812	China [171]	GO (n=190), GH (n=561)	P=0.003, OR=1.45
IL-16	rs4778889, rs1131445, rs4778641	China [280]	GO (n=136), GH (n=122)	Haplotype (C-T-C) P=0.013, OR=0.57
**Immune- modifying genes: other**				
CTLA4	A49G	UK [281]	GO (n=129), GH (n=172)	P=0.006, OR=2.1
CTLA4	C-318T	China [282]	GO (n=142), GH (n=121)	P=0.009, OR=0.51
ICAM-1	A1405G	Poland [283]	GO (n=108), GH (n=127)	P=0.003, OR= 1.8
PTPN12	rs1468682, rs 4729535	UK [284]	mild GO# (n=354), GH (n=366)	P=0.004, OR=1.41 P=0.006, OR=1.37
NFKB1	-94ins/del ATTG	Japan [223]	GO (n=123), GH (301)	P=0.009, OR=1.37
TLR-9	rs352140	Taiwan [285]	GO (n=200), GH (271)	P=0.03, OR=1,97 in male GD patients
CD86	rs_9831894	Taiwan [286]	GO (n=200), GH (271)	P = 0.0017

GO - Graves’ ophthalmopathy, GH - Graves’ hyperthyroidism without eye disease, HS – healthy subjects.

# Mild GO - NOSPECS score 2-4.

**Table 2.** Selected Genetic Markers Associated with the Clinical Course of Hyperthyroidism in Graves’ Disease

Locus	Polymorphism (allele)	Population	Studied group	Evaluated parameter
**Immune- modifying genes**				
HLA	HLA-DRB1*07	Denmark [287]	Juvenile GD (n=102)	Age of onset of GD
ICAM-1	G721A	Poland [283]	GD (n=235)	
PTPN22	C1858T	Poland [57]	GD (n=290)	
NFKB1	-94ins/del ATTG	Japan [223]	GD (n=424)	
CD40	C-1T	Taiwan [143]	GD (n=215)	
CTLA4	A49G	Japan [288]	GD (n=144)	Remission/relapse rate after ATD treatment
CTLA4	A49G	Japan [289]	GD (n=415)	
CTLA4	A49G	Taiwan [290]	GD (n=208)	
CTLA4	A49G	Turkey [146]	GD (n=97)	
TG	E33SNP	Taiwan [142]	GD (n=215)	
CXCL10	rs8878	Germany [291]	GD (n=228)	Severe course of GD#
CTLA4	A49G	Slovenia [292]	GD (n=67)	TPOAb and TgAb levels
TNF	C-857T	Japan [293]	GD (n=75)	TRAb level
**Thyroid-specific genes**				
TSHR	rs2239610	China [145]	GD (n=436)	FT4 and TRAb levels

ATD anti-thyroid drugs, TPOAb anti-thyroid peroxidase antibodies, TGAb anti-thyroglobuline antibodies, TRAb anti-TSHR antibodies.

# The cutoff for a severe course of GD was defined by the presence of Graves’ ophthalmopathy, recurrent hyperthyroidism, thyreotoxic crisis, large goiter and/or other autoimmune diseases.
